# Thyroidectomy in children^⋆,⋆⋆^

**DOI:** 10.1016/j.bjorl.2025.101642

**Published:** 2025-05-24

**Authors:** Flávio Carneiro Hojaij, Onivaldo Cervantes, Cindel Nogueira Zulino, Leonardo Dantas Pereira, Felipe Augusto Brasileiro Vanderlei, Marcio Abrahao

**Affiliations:** a Faculdade de Medicina da Universidade de São Paulo (FMUSP), LIM 02, São Paulo, SP, Brazil; b Universidade Federal de São Paulo (UNIFESP), Departamento de Otorrinolaringologia e Cirurgia de Cabeça e Pescoço, São Paulo, SP, Brazil; c Hospital das Clínicas da Faculdade de Medicina da Universidade de São Paulo (HCFMUSP), Cirurgia Geral, São Paulo, SP, Brazil; d Hospital das Clínicas da Faculdade de Medicina da Universidade de São Paulo (HCFMUSP), Cirurgia de Cabeça e Pescoço, São Paulo, SP, Brazil

**Keywords:** Thyroid gland, Thyroidectomies, Children

## Abstract

**Objective:**

Analysis of the surgical approach and postoperative outcomes of 59 pediatric patients submitted to thyroidectomy between 2003 and 2019.

**Methods:**

An outpatient postoperative follow-up of 59 patients aged 3–18 years submitted to thyroidectomy for different causes was performed, and immediate and late postoperative outcomes were analyzed. The operations were performed at a University Hospital as well as at Private Tertiary Hospitals.

**Results:**

The following postoperative outcomes were observed: three tracheostomies, 22 patients with transient hypoparathyroidism, eight patients with definitive hypoparathyroidism, and three patients with vocal fold paralysis.

**Conclusion:**

Thyroidectomy in children is a procedure with a higher complication rate and needs to be performed by a team of high-volume surgeons. This study focuses on the adverse events, so that those interested in the subject are aware of them.

**Level of evidence:**

Level III.

## Highlights

• 36% of pediatric patients presented with permanent hypoparathyroidism.
• Rate of vocal fold paralysis is 5% in this population.
• Rate of complications are higher than the expected adult patients.

## Introduction

Thyroid diseases are subject to clinical and surgical treatments, or a combination of both. The surgical approach should be considered in selected cases, such as nodules suspicious for malignancy and large and toxic goiters. In addition, prophylactic thyroidectomy is performed in children with genetic syndromes predisposed to malignancy. Surgical management of thyroid disease in children presents several peculiarities and has increasingly developed in recent years, although historically few studies conducted with this population have been published.^1^ The interest in conducting the present study arose from these reasons.

A literature review conducted in Sweden from a national registry of pediatric patients demonstrated that thyrotoxicosis was the main indication for total thyroidectomy in that country, followed by malignant neoplasms and other benign thyroid diseases.^2^ Hyperthyroidism accounts for 15% of the thyroid disease cases in this population, mostly attributed to autoimmunity (Graves’ disease).^3^ The vast majority of these patients were initially treated with antithyroid medications and, in the absence of remission (approximately five years of treatment) or in the presence of Adverse Events (AE), underwent Total Thyroidectomy (TT).^4^

According to the American Thyroid Association Guidelines Task Force on Pediatric Thyroid Cancer, new cases of thyroid cancer in people aged <20-years account for 1.8% of thyroid malignancies annually, but this incidence seems to be increasing.^5^

Papillary Thyroid Carcinoma (PTC) is the most common histological type of thyroid cancer in children as well as in the adult population. It is noteworthy that there is a growing tendency to detect metastasis at diagnosis in children compared with the adult population; however, children tend to respond better to therapy and present favorable prognoses.^6^

Exposure to radiation is also an important risk factor for developing malignant thyroid neoplasms. Reports from Belarus, after the Chernobyl accident, indicated clinical and molecular differences between patients exposed to ionizing radiation and those with ‘sporadic’ cancer.^7^

When discussing prophylactic surgery, Medullary Thyroid Carcinoma (MTC) and Multiple Endocrine Neoplasia (MEN) are mandatory references. Depending on the “Rearranged During Transfection” (RET) mutation ‒ RET mutation, a total thyroidectomy or even an association with emptying of the central compartment can be performed.^8^

According to the panorama of thyroidectomy in children, several complications may occur after TT, with hypocalcemia as the most common complication. Pediatric patients tend to have more perioperative complications in thyroid surgeries than adult patients: the younger the child, the longer the hospital stay and the association with serious procedural complications. Respiratory and infectious complications as well as bleeding are other possible medical aggravations of thyroid surgery in children.

A retrospective study conducted by Tuggle and collaborators on thyroid surgeries and examinations performed in the USA between 1999 and 2005 suggested that surgical outcomes were more satisfactory when these procedures were performed by high-volume surgeons (>30 cervical endocrine surgeries per year). The aforementioned study also showed an improvement in hospital cost and length of stay when procedures were performed by high-volume surgeons compared with those conducted by pediatric and general surgeons or otorhinolaryngologists.^9^

This study aimed to analyze the evolution of pediatric patients undergoing surgical procedure as a consequence of thyroid disorders and to report complications and AE observed in these patients, especially for those interested in the subject. A surgical team with experience in the procedure and the age range of patients may decrease the rates of postoperative complications1.

## Methods

**Fig. 1:**
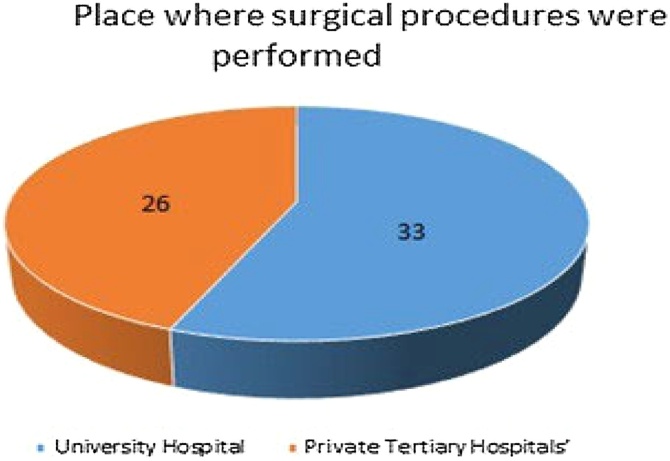
Place where surgical procedures were performed.

This study was based on patients operated by Head and Neck (H&N) Surgery teams in the presence of the same lead surgeon in a University Hospital (33 operations) and at Private Tertiary Hospitals (26 operations) from 2003 to 2019 (Fig. 1). A retrospective evaluation of these cases was conducted by simple analysis of medical records.

**Fig. 2:**
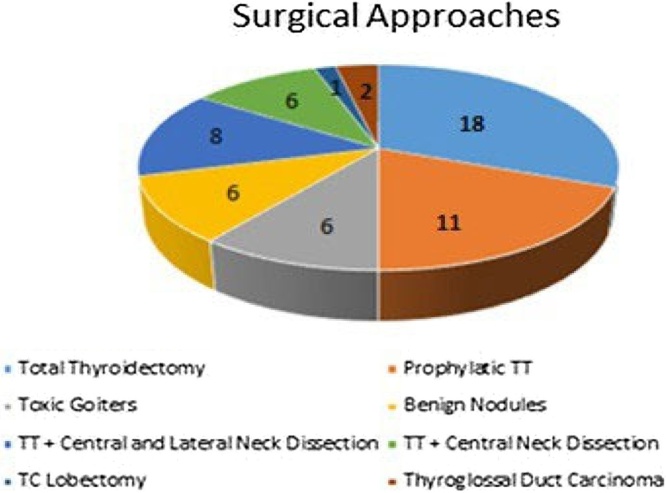
Surgical Approaches.

The following surgical approaches were conducted on 59 patients aged 2–18 years, including 13 children weighing <30 kg: 18TT, 11 prophylactic thyroidectomies, eight lateral Neck Dissections (ND) and central ND combined with TT, six TT associated with emptying of the central compartment, six thyroidectomies for toxic goiters, six thyroidectomies for benign nodules, two thyroidectomies for thyroglossal duct carcinoma, and one lobectomy for Thyroid Carcinoma (TC) (Fig. 2).

Intraoperative care was provided in all cases, e.g., adequate room temperature, delicate materials, thermal mattress, preservation of neurological structures, and focused neurological dissection (nerves). Nerve Monitoring (NM) was not performed in the vast majority of the patients (48), and advanced energy equipment was not used either. Therapeutic ND were indicated according to the guidelines.

Outpatient follow-up proceeding to post-operative evolution was performed for at least six months.

## Results

Only adverse results are informed in this study, as expected results do not need to be described.

**Fig. 3:**
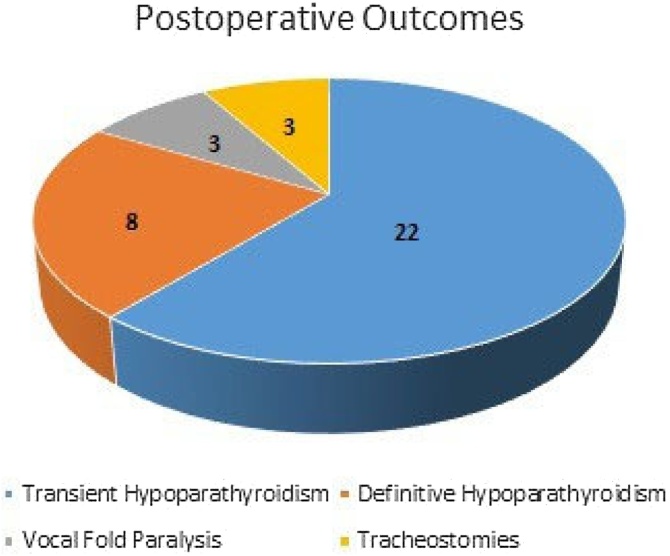
Postoperative Outcomes.

The following postoperative outcomes were addressed in the 59 patients operated and followed-up by the surgery teams: three tracheostomies were performed, 22 patients had transient hypoparathyroidism, eight individuals presented definitive hypoparathyroidism (requiring doses of calcium and vitamin D replacement after six months of follow-up), and three patients had Unilateral Vocal Fold Paralysis (UVFP) (within six months, postoperatively) (Fig. 3). Only one patient was surgically reopened for completing thyroidectomy after definition of the pathological outcome. No patients developed surgical wound infection, and there were no deaths in the analyzed period.

## Discussion

The younger the child, the longer the hospital stay and the occurrence of serious complications associated with the procedure. Thyroidectomies in adolescents and children weighing >30 kg use the operative technique, and have evolution and care similar to those in adults. In addition, higher percentages of lymph node metastasis, hypoparathyroidism and recurrence rates have been observed. In children weighing <30 kg, a larger number of respiratory complications and hypoparathyroidism have been verified. Such complications are associated with the soft tissue structures addressed during surgery, and are mainly found in patients submitted to ND. In these cases, the surgical technique needs to be revised with regard to the manipulation of these structures with the use of low-impact energies, such as advanced bipolar or harmonic scalpels, accustomed to the anatomy (Laryngeal Nerves [LN], parathyroid glands, and increased thymus in younger children).^1^

Regarding the study sample, 13 (22%) patients weighed <30 kg. TT was performed in 30% of the patients, and ND was required in 13.5% of them.

These numbers demonstrate the high complexity of the sample and the difficulty found in intra- and post-operative management.

Although rare, the existing literature varies substantially concerning the results of thyroidectomy in children.

In 2016, a study conducted with a Norwegian population reported 25% incidence of definitive hypoparathyroidism after TT10. A similar Swedish research (2018) found a rate of 7.3%.^2^ In the present work, an intermediate rate was obtained: 13.5%.

In the USA, a similar population-based study verified that, in a total of 1654 TT, 21 cases required tracheostomy, most of them in children aged <1-year. In the present study, three patients (5%) underwent postoperative tracheostomy, with two of these procedures conducted in children weighing <30 kg. In the same American study, 1.7% of the patients developed Vocal Fold Paralysis (VFP), whereas in this study, 3 (5%) patients evolved to this outcome.

The complication rates (hypoparathyroidism, need for tracheostomy and VFP) indicated in this study are higher than the series described in adults. Such complications can be explained by the complexity of the patients: pediatric age and its particularities, need for neck emptying, and difficulties related to children weighing <30 kg. It is also worth noting that these events occurred in the initial series, from 2003 to 2007, a fact that corroborates the need for experience not only in the procedure, but also in the management of patients in this age group.

## Conclusion

Thyroidectomy in children is a procedure that requires surgeons experienced in this type of surgery and in the management of patients in this age group. This fact can be justified by structural characteristics such as the soft tissue addressed and peculiarity of anatomical structures in children, the surgical pathophysiology, and the diligent technique to be employed in these patients.

Hypoparathyroidism was the most common complication observed in this series. Adverse Events (AE), such as the need for postoperative tracheostomy and Vocal Fold Paralysis (VFP), were also reported.

Although there are few studies addressing this specific topic in the literature, it was possible to observe that a team of high-volume surgeons can diminish the rates of postoperative complications observed in patients. Multicenter studies with larger samples may better explain the Brazilian condition regarding thyroid surgery in children.

## Financial support

None.

## Declaration of competing interest

The authors declare no conflicts of interest.
